# Subtype-specific survival and regeneration of retinal ganglion cells in response to injury

**DOI:** 10.3389/fcell.2022.956279

**Published:** 2022-08-12

**Authors:** Mary L. Tapia, Gabriel Nascimento-dos-Santos, Kevin K. Park

**Affiliations:** Department of Neurological Surgery, The Miami Project to Cure Paralysis, University of Miami Miller School of Medicine, Miami, FL, United States

**Keywords:** retinal ganglia cells (RGC), axon regeneration, ipRGCs, melanopsin ganglion cells, axon injury, optic nerve injury, axon growth

## Abstract

Retinal ganglion cells (RGCs) are a heterogeneous population of neurons that function synchronously to convey visual information through the optic nerve to retinorecipient target areas in the brain. Injury or disease to the optic nerve results in RGC degeneration and loss of visual function, as few RGCs survive, and even fewer can be provoked to regenerate their axons. Despite causative insults being broadly shared, regeneration studies demonstrate that RGC types exhibit differential resilience to injury and undergo selective survival and regeneration of their axons. While most early studies have identified these RGC types based their morphological and physiological characteristics, recent advances in transgenic and gene sequencing technologies have further enabled type identification based on unique molecular features. In this review, we provide an overview of the well characterized RGC types and identify those shown to preferentially survive and regenerate in various regeneration models. Furthermore, we discuss cellular characteristics of both the resilient and susceptible RGC types including the combinatorial expression of different molecular markers that identify these specific populations. Lastly, we discuss potential molecular mechanisms and genes found to be selectively expressed by specific types that may contribute to their reparative capacity. Together, we describe the studies that lay the important groundwork for identifying factors that promote neural regeneration and help advance the development of targeted therapy for the treatment of RGC degeneration as well as neurodegenerative diseases in general.

## 1 Introduction

Retinal ganglion cells (RGCs), which are the exclusive output neurons in the retina transmit a distinct set of parallel, highly processed visual information to target areas in the brain through their axons. These axons project to various brain regions including the lateral geniculate nucleus (LGN) in the thalamus and the superior colliculus (SC) in the midbrain, where information is conveyed to the higher visual processing centers. This is how we are able to perceive visual images of the world around us.

The eye is an effective model for studying central nervous system (CNS) injury and diseases. Since the retina and optic nerve extend from the diencephalon during embryonic development, they are an extension of the CNS. As such, the retina displays the characteristic properties of CNS neurons and offers an accessible and organized platform from which to study the CNS. The retina displays similar structural and functional features as the brain and spinal cord and responds similarly to injury. In fact, substantial knowledge about how axons respond after CNS trauma has emerged from optic nerve studies (Vidal-Sanz et al., 1987; Villegas-Perez et al., 1988; Keirstead et al., 1989; Benowitz and Yin, 2008; Berry et al., 2018). The eye also has limited interactions with the immune system and is surrounded by an assortment of barriers to blood, which are also characteristic features of the brain and spinal cord. Additionally, the anterior chamber of the eye is filled with fluid enriched aqueous humor that consists of anti-inflammatory and immunoregulatory mediators that are comparable to the cerebrospinal fluid that circulates throughout the brain and spinal cord parenchyma (Wilbanks and Streilein, 1992; Taylor and Streilein, 1996). Injury to CNS axons and resultant neuronal cell death are key features of many neurodegenerative diseases that can be modeled in the eye as injury to RGCs or their axons often cause functional loss, leading to blindness and other optic neuropathies (Ghaffarieh and Levin, 2012; You et al., 2013).

RGCs of the eye are a heterogeneous population of neurons having different morphological (i.e., cell soma size, stratification), physiological (e.g., some respond to movement while others respond to light or regulate circadian rhythm), molecular (e.g., having a varied enrichment of genes), and mosaic features (i.e., dendritic arborization). By combining these features, many RGCs have been classified into distinct types and different RGCs types have been shown to respond differently to injury or disease. After damage to the optic nerve, for instance, many RGCs die, and those that do survive usually fail to regrow axons and restore synaptic connections. Recently, genetic studies have identified genes that are differentially expressed in resilient and vulnerable RGC types to help elucidate the molecular mechanisms behind this disproportionate response. The ability to identify the ways in which diverse RGC subtypes are susceptible to injury or disease allows for the investigation of CNS disease mechanisms and the development of targeted therapies.

## 2 Retinal ganglion cell heterogeneity and classification of subtypes

RGCs have been well studied for their diversity, connections, signaling, and development. In mice, studies have identified more than 40 distinct RGC subtypes (Masland, 2012; Sanes and Masland, 2015; Rheaume et al., 2018; Tran et al., 2019). In this section, we provide a summary of RGC types that have been well established in the literature and shown to demonstrate a differential response to injury among other RGC types.

### 2.1 Intrinsically photosensitive retinal ganglion cells

The Intrinsically photosensitive retinal ganglion cells (ipRGCs) are a physiologically and morphologically heterogeneous population of cells that express the visual pigment melanopsin (encoded by the gene *Opn4*) and are intrinsically photosensitive. The expression of melanopsin gives ipRGCs the capacity for autonomous phototransduction, independent of rods and cones. ipRGCs comprise about 1% of the total RGC population in humans and can be distinguished by their large and sparse dendritic fields. This atypical class of ganglion cell photoreceptors project broadly throughout the brain and regulate a varied array of non-image forming visual functions, including pupillary light reflex, photoentrainment of our circadian rhythms, regulation of sleep/wakefulness, mood, and body temperature (Chen et al., 2011; Schmidt et al., 2011b). In addition, more recent studies point also to a role for ipRGCs in pattern vision (Schmidt et al., 2014; Sonoda and Schmidt, 2016).

In mice, there are six subtypes of ipRGCs (M1–M6) that have been identified to date. These subtypes can be distinguished by the size and intricacy of their dendritic arbors and by the degree of stratification achieved by their dendrites within the synaptic inner plexiform layer (IPL). M1 ipRGCs have dendrites that stratify in the outer limit of the OFF sublayer of the IPL. They also have a small somata and simple dendritic arbors (Baver et al., 2008; Schmidt and Kofuji, 2009; Berson et al., 2010). M2 ipRGCs stratify the innermost sublamina (within the ON sublayer) of the IPL. They have complex dendritic arbors and larger cell soma sizes than M1 type (Schmidt and Kofuji, 2009; Berson et al., 2010). M3 ipRGCs are bistratified cells that stratify onto both ON and OFF sublaminas; otherwise they are analogous to M2 type in soma size and dendritic arbor complexity (Ecker et al., 2010; Schmidt and Kofuji, 2011). Both M4 and M5 ipRGCs have dendrites that stratify in the ON sublayer of the IPL, and M4 has been shown to be synonymous with ON alpha RGCs (Ecker et al., 2010; Schmidt et al., 2014; Rheaume et al., 2018). M4/alpha RGCs have the largest somata of any identified ipRGCs and the largest dendritic arborization. In contrast, M5 types have small somata and highly branched “bushy” dendritic arbors (Ecker et al., 2010). M6 ipRGC have bistratified dendritic arbors that are small and highly branched, which laminate both ON and OFF sublayers of the IPL. They also have the smallest somata of any described ipRGC types (Quattrochi et al., 2019). ipRGC subtypes exhibit differential expression of melanopsin, with M1 type and M4-M6 types expressing the highest and lowest levels of melanopsin, respectively (Aranda and Schmidt, 2021).

Subtypes of ipRGCs also project to different target areas in the brain. A combination of genetic and neuronal tracing approaches have provided ipRGC projection maps that demonstrate the axonal projection targets for individual ipRGC types (Figure 2). M1 type ipRGCs mainly project to non-image forming retinorecipient areas in the brain, including the suprachiasmatic nucleaus (SCN) and olivary pretectal nucleus (OPN) to drive circadian photoentrainment and pupillary light reflex function, respectively (Hattar et al., 2006; Li and Schmidt, 2018). M4–M6 types project to image-forming targets including the SC and dorsal lateral geniculate nucleus (dLGN), whereas M3 ipRGCs axons have thus far only been shown to project to the SC (Güler et al., 2008; Estevez et al., 2012; Zhao et al., 2014). Uniquely, M2 ipRGCs axons project to both image forming and non-imaging retinorecipient brain targets (Baver et al., 2008).

Furthermore, ipRGC subtypes also exhibit differential responses to light. The variable nature of their photoresponses arises from the ratio of the intrinsic melanopsin response versus integration with photoreceptor cell circuitry as well as from the differences in morphology (i.e., dendritic stratification) between each subtype. Therefore, the duration, magnitude, and latency of responses to light differs in an intensity-dependent fashion among different ipRGC subtypes.

ipRGC subtypes identified in the mouse retina have also been documented in other mammalian species. In the rat retina all M1–M5 ipRGCs types have been identified, and their morphologies also match those of ipRGCs in mice (Li et al., 2006; Warren et al., 2006; Boudard et al., 2009; Estevez et al., 2012; Esquiva et al., 2013; Reifler et al., 2015). Diverse spontaneous spike rates were exhibited by different ipRGC subtypes in rats. Similar to observations in mice, M4 type had the most spikes while M1 type had the least spikes. In addition to the slow intrinsic photoresponses generated by rat ipRGCs, they also produced fast and synaptically driven photoresponses—response patterns which are also noted in mice (Reifler et al., 2015). In primates, ipRGCs stratifying into outer and inner laminates of the IPL have been identified in the retinas of marmosets, macaques, and humans (Hannibal et al., 2004; Dacey et al., 2005; Jusuf et al., 2007). ipRGCs in humans can also be characterized as in mice (Schmidt et al., 2011a) with M1–M4 types having been identified in human retina (Hannibal et al., 2017). As in mice, the dominant subtype found in humans was the M1 type, with a high density displaced in the inner nuclear layer (Baver et al., 2008; Ecker et al., 2010). Additionally, ipRGCs identified in mammalian species also contain the photopigment melanopsin. These findings suggest there is some conservation of ipRGC types and likely corresponding roles in behavior.

### 2.2 Alpha retinal ganglion cells

Among the main recognized RGC types are alpha RGCs. Alpha RGCs were first identified by Wässle and collaborators in their early studies performed in the cat retina (Cleland et al., 1975; Wässle et al., 1981; Peichl, 1991). Alpha RGCs are a distinct morphological class identified by their large cell bodies, large mono-stratified dendritic arbors, stout axons and dendrites, and high expression of neurofilament proteins (Boycott and Wässle, 1974; Peichl et al., 1987b). Alpha RGCs also share specific physiological properties, including a short latency response and axons with a faster conductance than most other RGCs (Cleland et al., 1975; Peichl and Wässle, 1981), which is why they are among the first to signal a new stimulus to the brain. In the mammalian retina, there are two subclasses of alpha cell which have opposite responses to a change in luminance: one is ON center and the other is OFF center.

Alpha RGCs are among the largest RGC types identified in the mouse eye. All alpha RGC subtypes have large dendritic field sizes (∼300 µm in diameter) and large soma diameters (>15 µm) with similar total dendrite lengths. All alpha RGCs dendrites are monostratified in thin bands within the IPL. Although alpha RGC types are morphologically indistinguishable when viewed from above (in-plan view) they have distinct levels of vertical dendritic stratification within the IPL. Since their laminar position sets restrictions on what signals can be received, this distinction further characterizes alpha RGCs into four distinct types based on their light responses: ON-sustained (ON-S), ON-transient (ON-T), OFF-sustained (OFF-S), and OFF-transient (OFF-T) (Murphy and Rieke, 2006; Margolis and Detwiler, 2007; Wyk et al., 2009; Krieger et al., 2017).

In addition to their characteristic large cell soma and neurofilament rich protein expression, all alpha RGC subtypes also share molecular features that differentiate them from non-alpha RGCs. All alpha RGCs express *Kcng4*, which encodes for voltage-gated K^+^ channel subfamily G member 4, and high levels of *Ssp1*, which encodes for secreted phosphoprotein osteopontin (Duan et al., 2015). Furthermore, all four alpha RGC types have a weak antagonistic surround, a large center receptive field, and lack of selectivity for a particular direction. All mouse alpha RGCs project to both primary retinorecipient areas in the brain, the SC and dLGN, providing substantial input at these central targets that plays an important role in visual processing (Dhande and Huberman, 2014).

Alpha RGCs have been identified in the retinas of over 30 different mammalian species, including cats, rabbits, rats, dogs, macaques, and humans (Boycott and Wässle, 1974; Wässle et al., 1975; Perry and Cowey, 1981; Rodieck et al., 1985; Peichl et al., 1987a; Peichl, 1989, 1992). For instance, the Y-cells observed in cats and the parasol ganglion cells noted in primates have large regularly spaced somata with large dendritic fields that branch in a mosaic-like pattern and share similar physical traits as those of rodent-characterized alpha RGCs (Crook et al., 2008; Wyk et al., 2009; Krieger et al., 2017). Thus, the morphology and features of alpha RGCs seem to be conserved across species of mammals despite their different ways of life and their different habitats. This conservation may suggest that alpha RGCs are a required ganglion cell type of the mammalian retina that fulfills a fundamental role required for visual function.

### 2.3 Direction-selective retinal ganglion cells

A subpopulation of RGCs known as direction-selective retinal ganglion cells (DSGCs) respond selectively to objects moving in particular directions. Notably, DSGCs can be distinguished from other RGCs by their unique expression of the gene *Cartpt* that encodes for the neuropeptide cocaine- and amphetamine-regulated transcript (CART) (Kay et al., 2011). DSGC types can either respond to both light onset and offset (ON-OFF) or just to light onset (ON) alone. These functional qualities give rise to four types of ON-OFF DSGCs (ooDSGCs) and three types of ON DSGCs (oDSGCs). ON-OFF DSGCs can detect motion in one of the four cardinal axes [dorsal (D-), ventral (V-), nasal (N-), or temporal (T-) direction] while ON DSGCs can only detect movement in the ventral, dorsal, and nasal directions (Oyster and Barlow, 1967). Advances in mouse genetic studies have also led to the discovery of an OFF DSGC type that detects upward motion in the visual scene (Kim et al., 2008).

The most highly studied population of DSGCs are ooDSGCs. The dendrites of these RGCs are bistratified and laminate both the ON and OFF sublaminate layers of the IPL where they receive synapses from starburst amacrine cells (the key source of inhibitory input) and glutamatergic interneurons (the key source of excitatory input) in circuits that produce their direction selectivity nature (Kay et al., 2011; Sanes and Masland, 2015).

Although most ooDSGCs project mainly to SC and dLGN, there are subtype-specific projection differences of ooDSGCs to their central targets in the brain (Figure 2B). N- and V- ooDSGC axons project to the dLGN where their dendritic arbors are partially segregated, and D- and V- preferring ooDSGCs axons specifically project to the nucleus of the optic tract (NOT) and medial terminal nucleus (MTN) (Kay et al., 2011).

### 2.4 Other select retinal ganglion cell types

There are also RGCs that have been classified based on their functionality. Local edge detectors or LEDs are a class of ganglion cell that responds selectively to luminance edges which is an essential feature needed to transform the visual scene into perceivable boundaries. These cell types were first detected in cat and rabbit retina, but there is also an evolutionary conserved cell type in the mouse retina that is morphologically and physiologically similar to LEDs known as W3-RGCs (Levick, 1967; Cleland and Levick, 1974; Kim et al., 2010; Zhang et al., 2012), making these RGCs an evolutionarily conserved visual channel. These cells have extensively branched arbors that stratify at the center of the IPL and represent about 13% of total RGCs near the visual field center which then taper off toward the periphery (Zhang et al., 2012). W3-RGCs are among the most numerous RGCs and among the smallest RGCs regarding cell soma and dendritic arbor size. There are two W3-RGC populations identified in the mouse, the W3B and the W3D population. W3B-RGC subtypes are sensitive to motion but not direction while W3D-RGCs remain physiologically uncharacterized (Zhang et al., 2012).

Some RGCs have been categorized based on their molecular expression. Junctional adhesion molecule B (JAM-B) is used to identify the mosaic arrangement of a population of OFF RGCs known as J-RGCs (Kim et al., 2008). J-RGC dendrites expand to the outer sublamina of the IPL, have asymmetric dendritic arbors that have a dorsal-to-ventral alignment across the retina, and respond only to stimuli that are moving in a soma-to-dendrite direction. Having strong asymmetric receptive fields, J-RGCs’ directional behavior is very different from that of DSGCs.

T- and F-RGCs are cell types defined by their expression of the transcription factors T-box brain transcription factor 1 (*Tbr1*) and forkhead/winged-helix domain protein (*Foxp2*), respectively. *Tbr1* is expressed in four morphologically distinct OFF-laminating RGCs: J-RGCs, alpha-OFF-S, Tbr1-S1, and Tbr1-S2 which co-express brain-specific homeobox/POU domain protein 3B (*Brn3b*)*, Opn4, Brn3c,* and Calbindin 2 (*Calb2*)*,* respectively (Liu et al., 2018; Kiyama et al., 2019). F-RGCs, on the other hand, comprise a pair of small and numerous direction-selective RGCs, known as F-miniON and F-miniOFF, and a pair of bigger, less abundant, direction non-selective RGCs, known as F-midiON and F-midiOFF. In the mouse retina, T-RGCs comprise ∼11% of total RGCs while F-RGCs comprise >20% of total RGCs (Rousso et al., 2016). F-RGCs also contribute to visual perception (as do T-RGCs) as their axonal projections largely bypass non-image forming retinorecipient targets (including the SCN, MTN, and OPN) and terminate onto the dLGN and SC visual processing centers (Rousso et al., 2016).

We note that in this article, we limited our description to those RGC types whose injury responses are known. More exhaustive description on the various RGC types can be found in several excellent research and review articles (Sanes and Masland, 2015; Krieger et al., 2017; Rheaume et al., 2018; Laboissonniere et al., 2019).

## 3 Resistant and susceptible retinal ganglion cell subtypes after optic nerve crush injury

RGC death occurs within a few days following optic nerve crush (ONC) injury. When death-promoting cascades end up overshadowing expression of proteins that promote cell survival and regrowth, RGC death occurs (likely owed to Atf4 and caspases- mediated apoptosis) (Herdegen et al., 1993; Koistinaho et al., 1993; Robinson, 1994; Hu et al., 2012). Despite insults being widespread, RGCs come to their demise at different time-points with the majority of RGCs dying within 2 weeks after axonal injury close to the optic disc. In the optic nerve, the portion that is distal to the injury site undergoes Wallerian degeneration, which is characterized by an initial phase of axonal stability followed by rapid degeneration, blebbing and fragmentation of the residual axon, microtubule dismantling, and phagocytic clearance of the lesion site (Waller, 1850; Coleman, 2005). Axonal degeneration after ONC injury was found to be significantly delayed by allelic expression of slow Wallerian degeneration (Wld^S^) and sterile alpha and TIR motif containing 1 (Sarm1) deficiency (Mack et al., 2001; Fernandes et al., 2018). The degenerative pathways affected by these molecules are intrinsic to the axon and are molecularly distinct from the mechanisms that govern somatic RGC death, which are regulated by Bax-induced apoptotic pathway (Libby et al., 2005; Howell et al., 2007; Beirowski et al., 2008). Although some RGCs display strong resistance to axon injury, axons from these surviving RGCs mostly fail to regenerate beyond the site of injury.

Distinct RGC types have been shown to display differential responses to injury and disease. Some types of RGCs exhibit a selective resilience to injury while others are vulnerable and quickly die. Early studies in cats, for instance, showed that alpha RGCs persist after axotomy and have a greater propensity to regenerate their axons than other RGC types (Watanabe and Fukuda, 2002). Comparable studies done in mice have also shown that alpha RGC types preferentially survive axotomy. Additionally, when promoting axon regeneration by suppression of phosphatase and tensin homolog (Pten), Duan et al. (2015) found that alpha RGCs accounted for nearly all regenerating axons in this paradigm. The authors attribute this differential response of alpha RGCs to their characteristic high levels of mammalian target of rapamycin (mTOR) signaling, selective expression of osteopontin, and expression of insulin-like growth factor 1 (Igf1) receptors. In a later section we lay out the unique molecular characteristic of RGC types, specifically those shown to be either regenerative or susceptible after injury.

**FIGURE 1:**
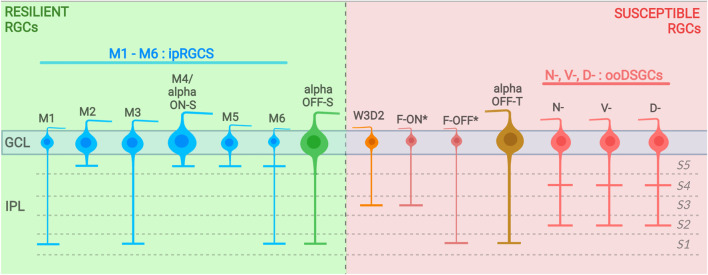
Resilient and susceptible RGC subtypes and their dendritic stratification into the IPL (with sublaminae divided into S1–S5). Resilient RGC subtypes are displayed on the left (background in green) and susceptible subtypes are displayed on the left (background in red). (*) includes F-midi and F-mini types. GCL, ganglion cell layer; IPL, inner plexiform layer.

**FIGURE 2:**
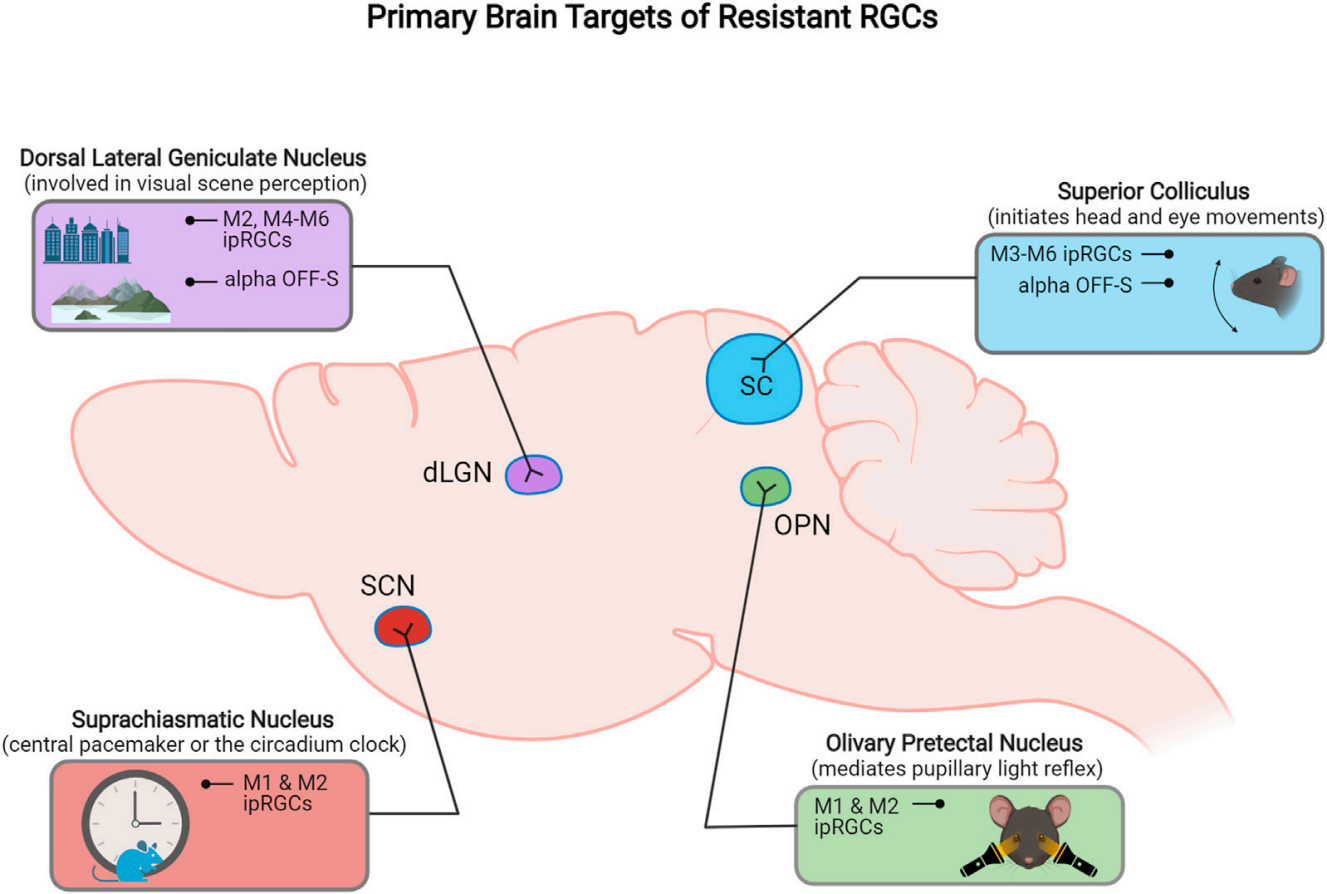
Schematic representation of the primary retinorecipient areas innervated by different resistant RGC subtypes.

**FIGURE 3:**
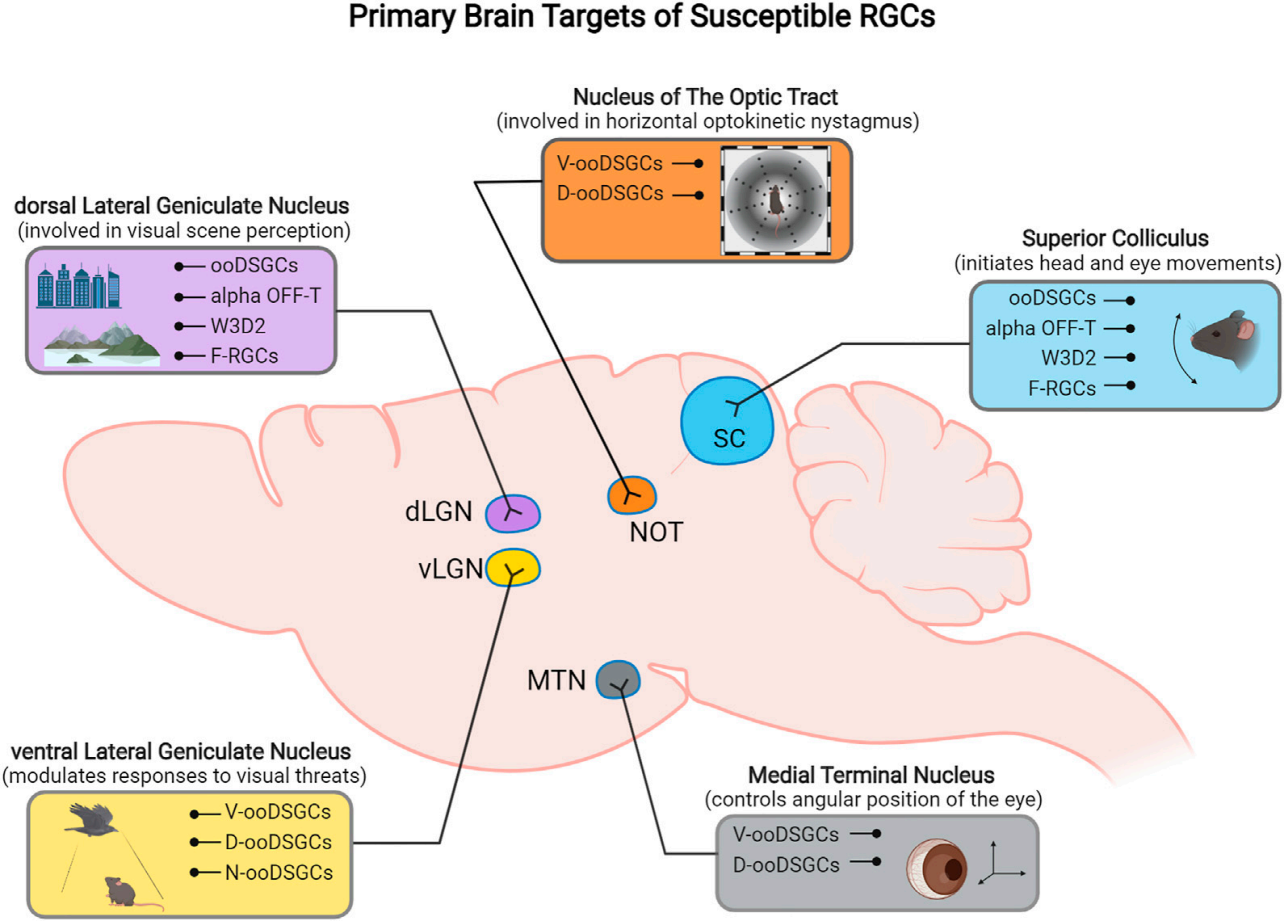
Schematic representation of the primary retinorecipient areas innervated by different susceptible RGC subtypes.

Previous studies have inconclusively identified several RGC types as resilient or susceptible to injury, but advances in cell transcriptomic profiling using transgenic mouse lines, with a greater sensitivity toward discriminating different RGC types, have improved this classification. Recently, Tran et al. (2019) performed high-throughput single-cell RNA-seq (scRNAseq) on RGCs and generated an atlas of adult mouse RGC types (46 in total). The authors used these data as groundwork to track RGC type-specific responses to injury. In this section and throughout this article, we describe the current RGC subtypes classified as ‘resistant’ and those classified to be 'susceptible' to injury identified in that study and others (Figures 1, 2, 3).

### 3.1 Resistant retinal ganglion cell subtypes after optic nerve crush injury

#### 3.1.1 Intrinsically photosensitive retinal ganglion cells (M1–M6)

ipRGC subtypes have been previously studied using various approaches that mainly rely on melanopsin expression (Li et al., 2016). However, these methods fall short in detecting subtypes that express low levels of melanopsin. Furthermore, melanopsin expression levels also tend to fluctuate in response to environmental and physiological changes, just as after injury (Sci, 2009; Nadal-Nicolás et al., 2015). For this reason, many previous studies involving ipRGCs primarily focused on M1–M3 subtypes, which express moderate to high levels of melanopsin. Recent work uses various Cre driver mouse lines that can track ipRGCs faithfully (Chen et al., 2011). Since these Cre driver lines have either Cre-recombinase or tamoxifen-inducible Cre gene inserted into the Opn4 locus, this is a more reliable technique to track all ipRGC subtypes, thereby considerably improving detection of M1–M6 ipRGC subtypes in studies of survival and regeneration. Previous and recent studies have shown ipRGCs to demonstrate some regenerative capacity to a variety of insults, including mitochondrial optic neuropathy, and transection of mouse or rat optic nerve (Park et al., 2008; Müller et al., 2014; Morgia et al., 2016). Bray et al. (2019) labeled ipRGCs using inducible and non-inducible Cre lines and found that ipRGCs have significantly higher survival rates compared to other RGCs following ONC. This is consistent with a previous study performed in mice where melanopsin expressing RGCs (42% survival) were shown to have a ∼3 fold increase in survival compared to non-melanopsin containing RGCs (11% survival) at 1 month post-axotomy (Robinson and Madison, 2004). In rats, melanopsin + RGCs also represent the largest population (∼82%) of all surviving RGCs at 2 months post-injury (Müller et al., 2014). According to the literature, only about 20% of RGCs survive at 2 weeks following ONC procedure (Park et al., 2008; Duan et al., 2015). At this time-point, Duan et al. showed that M1 and alpha RGCs are the primary residual population to survive the deleterious conditions. Here, M1 RGCs accounted for 11% of all surviving RGCs, and over 70% of total M1 types survived the insult. Tran et al. (2019) later confirmed that all ipRGCs subtypes (M1–M6) are resilient to injury in their scRNAseq profiling study. Not only were these cells impervious to death but they also maintained their physiological and morphological characteristics after injury. All resistant RGC types in their study, including ipRGCs, demonstrated similar firing rates during the 14-day duration of their physiological assessment. ipRGCs and other resilient types were also shown to retain their dendritic area and arbor complexity up until 14 days after axotomy. This is remarkable since, about 1 week after ONC, most RGCs typically experience a decrease in neurite outgrowth and dendritic complexity compared to uninjured RGCs (Kalesnykas et al., 2012).

After injury to the mouse optic nerve, <1% of surviving RGCs axons can spontaneously regenerate and extend past the site of damage. However, RGC axons can be induced to regenerate in a growth permissive environment, as noted in early studies, where a peripheral nerve is grafted onto a cut optic nerve stump and induces re-growth of RGC axons into the graft (Villegas-Perez et al., 1988; Watanabe et al., 1993). Although this technique promoted ipRGC survival, it failed to increase regeneration of their axons (Robinson and Madison, 2004). Later studies using genetic manipulation techniques such as deletion of Pten, suppressor of cytokine signaling-3 (Socs3), and Klf transcription factors, or overexpression of growth-promoting factors such as ciliary neurotrophic factor (Cntf) and Igf1 also successfully promoted regeneration of injured RGC axons (Cui et al., 1999; Park et al., 2008; Moore et al., 2009; Smith et al., 2009; Dupraz et al., 2013). At 6 weeks post-injury, Bray et al. (2019) observed a small number of spontaneously regenerating axons, of which 35%–50% consisted of ipRGCs. They also observed the regenerative ability of ipRGCs in various regenerative promoting paradigms. Treatment with adeno-associated virus (AAV)-Cntf demonstrated a substantial regeneration of ipRGC axons. With their inducible Opn4 Cre line, 15% of regenerated axons correspond to ipRGCs. Considering that ipRGCs represent only 0.5% of total RGCs, their axons constitute a considerable portion of regenerating axons. ipRGCs were also shown to be protected from cell death and demonstrated high regenerative ability after Pten ablation compared to the wildtype controls. These findings demonstrate that ipRGCs are more likely to regenerate than many other RGCs under some regenerative conditions. However, which ipRGCs subtypes make up the regenerative axon population has yet to be determined.

Although the molecular basis of ipRGC survival and regeneration has yet to be elucidated, studies that were dedicated to defining these mechanisms have identified several genes that play important roles for these processes in ipRGCs. Transcriptional profiling studies have previously detected *Opn4*, T-box brain 2 (*Tbr2* or *Eomes*), and *Igf1* to be abundantly expressed in ipRGCs (Siegert et al., 2012; Macosko et al., 2015). Although ectopic overexpression of melanopsin promotes axon regeneration (Li et al., 2016), Bray et al. (2019) demonstrated that melanopsin is not required for the induction or sustained regeneration of ipRGCs after injury. In accordance with a previous study (Mao et al., 2014), they demonstrate that Tbr2 is important for the maintenance of ipRGCs in uninjured adult mice. Additionally, they show that Igf1 is also required for ipRGC maintenance but that conditional knock-down of either Tbr2 or Igf1 does not eradicate regeneration of ipRGC axons following ONC injury. Recently, Abed et al. (2022) used a *Tbr2* floxed transgenic line to knock-down expression of Tbr2 in the adult mouse retina. While Tbr2 ablation led to a reduction of melanopsin expression in ipRGCs it did not cause cell death, nor did it affect dendritic stratification or axonal projection pattern morphology. Ultimately, they concluded that Tbr2 expression is expendable for ipRGC survival.

#### 3.1.2 Alpha retinal ganglion cells (OFF-sustained alpha retinal ganglion cells and ON-sustained alpha retinal ganglion cells/M4 intrinsically photosensitive retinal ganglion cells)

Currently, two types of alpha RGC can be classified as resilient. Duan et al. (2015), used a transgenic mouse line where Cre recombinase is incorporated into the locus encoding Kcng4 which provided them with selective genetic access to alpha RGCs. In these studies, the authors found that more than 80% of alpha RGCs survive these injurious conditions, accounting for 23% of total surviving RGCs. This preferential survival does not correspond to a delayed cell loss, as these alpha RGCs are still preferentially spared and comprise 25% of all surviving RGCs at 4 weeks post-crush. More recently, Tran et al. (2019) further extended these findings and concluded that two alpha RGC subtypes exhibit resistance to cell death. These resistant alpha RGCs are ON-sustained (ON-S) and OFF-sustained (OFF-S) alpha RGCs, a result that is consistent with their *in vivo* cell recording findings that RGCs with sustained responses survive about 3-fold greater than transiently responding RGCs.

Kcng4-YFP^+^ alpha RGCs were also found to exhibit substantial regeneration in Pten ablated mice following optic nerve injury, with axons regenerating at least 0.5 mm past the crush injury site (Duan et al., 2015). Alpha RGCs were shown to have high levels of mTOR activity, illuminating this regenerative behavior. Selective regeneration of alpha RGCs is also seen when osteopontin, which can stimulate mTOR activity, is introduced in combination with Igf1 or brain derived neurotrophic factor (Bdnf) in a manner that is as effective as Pten suppression. These characteristics further elucidate the regenerative ability of most alpha RGCs. It is unknown, however, whether axonal regeneration in this study was restricted to ON-S and OFF-S alpha RGCs subtypes.

Although most alpha RGCs demonstrate an intrinsic ability to regenerate their axons after ONC injury with deletion of Pten, this subtype is shown to have an altered response to this regenerative capacity when exposed to stromal cell-derived factor-1 (Sdf1) (Xie et al., 2022). In an inflammation-induced regeneration model by Xie et al. (2022), Sdf1 was shown to augment the effects of regeneration stimulating oncomodulin (Ocm). In this study, Sdf1 was shown to induce regeneration of RGC subtypes other than alpha RGCs. In fact, they show that exogenous administration of Sdf1 combined with deletion of Pten reduces the regenerative response of alpha RGCs, despite an increase in survival of these cells. Sdf1 shifts the regenerative pool from alpha RGCs to non-alpha RGCs, thereby dissociating the effects of Pten ablation on the survival and regeneration of alpha RGCs. Similar results were seen in another study where overexpression of Sox11 transcription factor was found to not just induce regeneration of non-alpha RGCs but specifically kill alpha RGCs even when administered in combination with Pten deletion. Moreover, co-treatment with Bcl-2 also failed to protect alpha RGC loss induced by Sox11 (Norsworthy et al., 2017). These results demonstrate that different growth-promoting interventions differentially affect survival and/or regeneration of distinct neuronal cell subtypes.

### 3.2 Highly susceptible retinal ganglion cell subtypes after optic nerve crush injury

#### 3.2.1 ON-OFF direction-selective retinal ganglion cells (all subtypes)

Previously, BAC transgenic HB9:GFP mice were used to track ventrally tuned ON-OFF ooDSGCs and detect their survival and regenerative capabilities following ONC procedure (Duan et al., 2015). Accordingly, it was demonstrated that few if any ooDSGCs survive at 2 weeks post-injury, and if any did survive, they fail to undergo axonal regeneration. In line with this finding, scRNAseq study observed that among all DSGCs, ooDSGCs are the most susceptible to injury. Interestingly, resistance did not vary between ON or OFF DSGC types yet ON-OFF DSGCs were much more susceptible to injury regardless of the feature selectivity (i.e., nasal, dorsal, ventral, or temporal preferring). Since ooDSGCs have transient responses, these findings are also consistent with the higher vulnerability exhibited by transient responding RGCs compared to sustained responding RGCs. Based on scRNA-seq-derived survival kinetics, it was also shown that N-, V-, and D-ooDSGCs types are among the susceptible RGCs. Additionally, this study observed a RGC cluster (assigned as “C24” cluster in the scRNAseq study) to be vulnerable to injury. Since this cluster is transcriptionally proximate to V- and D-ooDSGCs and their dendrites laminate the S2/S4 regions of the IPL, similar to ooDSGCs, this cluster was suggested to be the temporal-preferring (T-) ooDSGCs type (Peng et al., 2017; Tran et al., 2019). Although ooDSGCs are highly vulnerable to death, they surprisingly maintain their functional traits. The firing rates and orientation and direction-selectivity indices of these and other susceptible RGCs (dying between day 3 and 5 post-ONC) remain mostly unchanged, suggesting that activity levels are maintained just until before these RGCs undergo death (Tran et al., 2019).

To evaluate regeneration of ooDSGC axons, the optic nerves of HB9-GFP mice were crushed 2 weeks following Pten inhibition but the authors did not observe any regeneration of ooDSGCs in the optic nerve, distal to the site of injury (Duan et al., 2015). A later study performed ONC on HB9:GFP; Bax^−/−^ mice to test the regenerative ability of ooDSGCs when they are prevented from dying. Although the absence of pro-apoptotic gene Bax did halt ooDSGC death, no ooDSGCs axons were found to regenerate past the lesion site (Bray et al., 2019). Thus, ooDSGCs are incapable of undergoing axonal regeneration independent of their vulnerability to cell death.

#### 3.2.2 OFF-T alpha retinal ganglion cells

Duan et al. (2015) used transgenic mice (Kcng4-YFP) to label all alpha RGCs and observe their survival following ONC. Histological cross-sections through the retina revealed three surviving alpha RGC subtypes which were distinguished by their dendritic lamination in the IPL. These included ON-transient, OFF-transient, and OFF-sustained alpha RGCs. It was further described that OFF-T alpha RGCs exhibit a vulnerability that is similar to ooDSGCs (Tran et al., 2019). Thus, unlike other alpha RGCs, OFF-T alpha RGCs seem to be susceptible to death after ONC injury. Additionally, it was observed that while most susceptible RGCs retain their dendritic area until 4 days after injury, alpha OFF-T RGCs demonstrate a significant decrease in their dendritic arbor complexity.

#### 3.2.3 W3-retinal ganglion cells (W3D3 subtype)

A TYW3 mouse line with a YFP reporter (a conditional but not inducible Cre transgenic mouse line) (Kim et al., 2010) was used to identify W3-RGCs and evaluate their response to axotomy. The survival rates of YFP-expressing W3 RGCs (25.9% ± 2%) were found to be considerably lower than the total RGC population (33.5% ± 0.8%) at 7 days post-injury (Yang et al., 2020). Using the same mouse line, only intermediate survival of W3-RGCs (∼10%) was seen at 2-weeks post-ONC (Duan et al., 2015). Later, Tran et al. (2019) profiled single cells from the TYW3 mouse line where a subset of S3-laminating RGC dendrites were labeled with YFP and revealed that W3D2-RGCs are among the most vulnerable cell types classified in their survival groups. W3-RGC types also demonstrated no visible regeneration after administration of AAV-short hairpin RNA (shRNA) against Pten (Duan et al., 2015).

#### 3.2.4 F-retinal ganglion cells (all subtypes) and provisionally N-retinal ganglion cells (all subtypes)

All four known F-RGC types are shown to be susceptible to injury, including a newly identified fifth type (Tran et al., 2019). In particular, F-midi-RGCs have been identified as the most susceptible RGC subtype among all other identified RGCs. To date, the regenerative capacity of this cell type has yet to be evaluated. Based on transcriptional likeness and molecular markers, the study by Tran et al. (2019) was also able to identify a new RGC subclass from their dataset. This RGC type is conditionally labeled as N-RGCs and consists of eight subtypes in total. In this group, seven of eight types are apparently novel and possibly share cellular characteristics. At 14 days post-crush, all N-RGC subtypes survived poorly.

## 4 Molecular markers used to classify resilient and susceptible types of retinal ganglion cells

### 4.1 Resilient retinal ganglion cell type markers

#### 4.1.1 Intrinsically photosensitive retinal ganglion cells (M1–M6)

While detection of melanopsin expression has been a useful marker for most ipRGCs, this genetic identifier alone cannot distinguish individual ipRGC subtypes. ipRGC subtypes can primarily be identified by expression of both melanopsin and Tbr2. Tbr2 precedes the expression of melanopsin during neurogenesis and as previously mentioned, was found to be required for the preservation of melanopsin expression. Additionally, Mao et al. (2014) found that expression of melanopsin was restricted to Tbr2-expressing RGCs. Thus, they concluded that Tbr2 is a reliable predictor of ipRGCs. Subtypes of ipRGCs can be distinguished by specific markers. For instance, M1 ipRGCs can be distinguished by expression of adenylate cyclase activating polypeptide 1 (*Adcyap1*) and the absence of the neuromedin B (*Nmb*) gene while an additional M1 type can be identified by the co-expression of *Adcyap1* and *Nmb* (Tran et al., 2019). M2 subtype can be identified by the presence of *Spp1* and T-box transcription factor (*Tbx20*), known to be selectively expressed in ipRGCs and greatly enriched in M2 over other ipRGC types (Berg et al., 2019). M4/alpha ON-S RGCs were found to be distinguished by the expression of *Spp1* and interleukin one receptor accessory protein like 2 (*Il1rapl2*) while all other ipRGC types by serpin family E member 2 (*Serpine2*) and cadherin related family member 1 (*Cdhr1*) (Tran et al., 2019).

#### 4.1.2 Alpha retinal ganglion cells (OFF-sustained alpha retinal ganglion cells and ON-sustained alpha retinal ganglion cells/M4 intrinsically photosensitive retinal ganglion cells)

All alpha RGCs express *Spp1* (Duan et al., 2015). OFF-S alpha RGCs express *Tbr1* (Liu et al., 2018) and can be distinguished from other alpha RGCs by their expression of *Spp1* and FES proto-oncogene, tyrosine kinase (*Fes*) (Tran et al., 2019). Alpha ON-S/M4 RGCs uniquely express *Spp1* and *Il1rapl2*, as stated above.

### 4.2 Susceptible retinal ganglion cell type markers

#### 4.2.1 ON-OFF direction-selective retinal ganglion cells (all subtypes)

Initially revealed by microarray analysis, neuropeptide CART, was recognized to exclusively label all ooDSGCs distinguishing them from other RGCs (Kay et al., 2011). Cadherins have been shown to play a role in generating appropriate connectivity in the circuit formation of DSGCs. Although, cadherin 6 (*Cdh6*) is selectively expressed by D-, V-ooDSGCs, as well as some starburst amacrine cells, a more suitable marker to recognize D-/V-ooDSGCs was found to be collagen 25a1 (*Col25a1*) (Duan et al., 2018). N-ooDSGCs express transcription factor neuronal differentiation- 2 (*Neurod2*) (Tran et al., 2019), which is known to play a role in the specification of RGC subtypes. Additionally, *Mmp17* is differentially expressed in these cells and is a good indicator of N-ooDSGCs (Kay et al., 2011).

#### 4.2.2 OFF-T alpha retinal ganglion cells

Osteopontin is a typical marker for all alpha RGCs. Trophoblast glycoprotein (*Tpbg*) was previously shown to be expressed in a subtype of RGCs termed ON midget RGCs, which is equivalent to a population of alpha RGCs in mice (Peng et al., 2019). Detection of *Spp1* and *Tpbg* combined was later shown to be a reliable indicator of susceptible OFF-T alpha RGCs in their scRNA study (Tran et al., 2019).

#### 4.2.3 W3-retinal ganglion cells (W3D3 subtype)

The scRNAseq study found W3B, W3D (D1-3), F-mini-ON, F-mini-OFF, and T-RGC-S2 subtypes to express the integral membrane protein trafficking regulator of GLUT4 (SLC2A4) 1 (*Tusc5*). However, W3D3 subtypes were specifically identified by expression of prokineticin receptor 1 (*Prokr1*) which was observed to be differentially expressed in this subtype and validated by fluorescence *in situ* hybridization (FISH) (Tran et al., 2019).

#### 4.2.4 F-retinal ganglion cells (all subtypes) and provisionally N-retinal ganglion cells (all subtypes)

As stated earlier, F-RGCs are characterized as such by their expression of *Foxp2*. The combinatorial expression of *Foxp2* and the Brn3 transcription factors (*Brn3a-c*) initially helped differentiate these into four discrete subtypes that vary in cell size, dendritic lamination, and functional responsiveness (Rousso et al., 2016). Molecular markers specific to the detection of each of these subtypes were later discovered. F-midi-OFF were specifically identified by the presence of *Foxp2* and absence of *Brn3c* and calsenilin (*Kcnip3*), while expression of *Foxp2* and cyclin dependent kinase 15 (*Cdk15*) identified F-midi-ON subtypes. F-mini types, which were also shown to express *Tusc5* as stated above, could also be further distinguished by specific molecular markers. F-mini-ON subtype was identified by combined expression of *Foxp2* and iroquois homeobox 4 (*Irx4*) (Tran et al., 2019). F-mini-OFF subtype was recognized by phosphodiesterase 1A (*Pde1a*) expression, which was also previously observed by another group (Rheaume et al., 2018).

Provisionally named N-RGCs are a novel uncharacterized RGC type, which are vulnerable to injury. These cells are found to be closely related and co-express the transcription factors *Neurod2* and SATB homeobox 2 (*Satb2*) (Tran et al., 2019)*.*

## 5 Resistance and susceptibility of retinal ganglion cell subtypes in glaucoma and other animal models of injury

Recent findings demonstrate a variation in RGC susceptibility toward different types of neuropathy. Studies have presented valuable insights into the molecular mechanisms of RGC death in retinal diseases suggesting various mechanistic processes. RGC death occurs in many eye-blinding pathologies, including traumatic optic neuropathy (TON), Leber hereditary optic neuropathy (LHON), multiple sclerosis and glaucoma. In the case of TON, its pathogenesis seems to be multifactorial as there are several proposed mechanisms of RGC death, including deprivation of neurotrophic factors due to axonal transport failure, mitochondrial dysfunction, excitotoxicity, apoptotic signal cascade, reactive gliosis, loss of synaptic connectivity, and oxidative stress (Lebrun-Julien and Polo, 2008; Bessero and Clarke, 2010; Joshi et al., 2011; Almasieh et al., 2012). Glaucomatous pathology is characterized by an increase in intraocular pressure (IOP) followed by subsequent hypoxia of the optic nerve head and ischemia of the eye. This event causes RGC death from glutamate-induced excitotoxicity, an increase in inflammatory proteins, energy deprivation and dysfunction in the transport of trophic factors culminating in a blockage of retrograde and anterograde axonal transport and eventual loss of visual function. RGC death by apoptosis in glaucomatous animals was also documented by several studies (Kuehn et al., 2005; Almasieh et al., 2012; Tezel, 2013; Liberatore et al., 2017; Evangelho et al., 2019). Understanding the characteristic responses of RGC types to chronic vs. acute injuries may provide mechanistic correlations between RGC types and their susceptibility to distinct pathologies.

Most studies have addressed how diverse RGC subtypes respond to an acute injury to their axons (i.e., ONC or transection injury). However, the fate of RGC subtypes can vary depending on the type of insults. A recent paper compared the susceptibility of three different RGC subtypes (alpha RGCs, DSGCs, and ipRGCs) using both acute (ONC) and chronic glaucoma (polystyrene microbeads) in rats to address a possible differential response among the two models (VanderWall et al., 2020). Both Smi32+ alpha RGCs, Cart+ ooDSGCs, and Fstl4+ ON-DSGCs displayed high susceptibility to ONC. Alpha RGCs were resilient whereas DSGCs were highly susceptible to IOP increase. Tbr2+ ipRGCs were resilient to ONC, but susceptible to elevated IOP. Opn4+ ipRGCs displayed a high resilience to both acute and chronic insults. Others have shown that ipRGCs do not undergo significant apoptosis or morphological changes after chronic ocular hypertension by photocoagulation of the episcleral veins until at least 12 weeks post-intervention (Li et al., 2006).

More recently, in a mouse model of secondary congenital glaucoma caused by a spontaneous mutation of the SH3 and PX domains 2B (*Sh3pxd2b*) gene, it was shown that ooDSGCs have high susceptibility to glaucoma. On the other hand, ipRGCs were more resilient to death (Daniel et al., 2019). Others have shown that different ipRGC subtypes respond differently to an ocular hypertension (OHT) induced chronic model of experimental glaucoma. Eight to 9 weeks after photocoagulation of the trabecular meshwork, M4 ipRGCs displayed a 25% decrease in cell number while M1 ipRGCs displayed almost no cell loss, demonstrating higher resilience. Accordingly, there were only minor changes in behavioral functions regulated by the M1 ipRGCs, namely circadian re-entrainment and circadian rhythmicity. This study demonstrated that different ipRGC subtypes differ in their response to IOP increase (Gao et al., 2022).

In a laser-induced mouse model of glaucoma, photocoagulation had distinctive effects on the survival and functionality of different alpha RGC subtypes. While OFF-transient alpha RGCs were susceptible, ON- and OFF-sustained cells were more resilient to death. Accordingly, OFF-transient cells displayed a decrease in the spontaneous activity and receptive field size, which was not observed in the ON- and OFF-sustained alpha RGCs (Ou et al., 2016).

It is less clear whether susceptibility varies greatly among the different RGC types under other degenerative conditions including retinal ischemia, mitochondrial optic neuropathy, demyelinating diseases (e.g., multiple sclerosis) and traumatic brain injury (Hobom et al., 2004; Harper et al., 2021; Khan et al., 2021). A recent study reported that susceptibility of RGCs to N-methyl-D-aspartate (NMDA) excitotoxicity varies significantly among select RGC types, in which the alpha RGCs were the most resistant type while the J-RGCs were the most sensitive cells (Christensen et al., 2019), indicating that alpha RGCs are resistant to glutamatergic excitotoxicity.

There is also information on the susceptibility of defined RGC types in different human diseases. A study described the preservation of the light response conduction by the retinohypothalamic tract in patients with LHON and optic dominant atrophy (ODA). Patients were submitted to a melatonin suppression test that displayed a significant light-induced suppression of melatonin plasma levels in both control and visually impaired subjects, suggesting that ipRGCs are still competent to detect light and conduct the light stimulus to target areas in the brain (Morgia et al., 2010). On the other hand, studies conducted in Alzheimer’s disease patients showed a correlation between the reduction of nerve fiber layer thickness and abnormal circadian functional with significant loss of ipRGCs (Morgia et al., 2016). It was shown that patients with glaucoma frequently have an abnormal pupillary light reflex (PLR) (Feigl et al., 2011; Chang et al., 2013; Gracitelli et al., 2014; Kuze et al., 2017), suggestive of ipRGC degeneration. A psychophysical study using various sinusoidal gratings aimed at measuring parvocellular (P) ganglion cells and magnocellular (M) ganglion cells indicated a selective deficit in M ganglion cell density over P ganglion cell density in glaucoma patients (Anderson and O’Brien, 1997). However, a later study showed the reduction in contrast sensitivity to stimuli that isolate the magnocellular pathway was not significantly different compared with the reduction in contrast sensitivity to stimuli that isolate the parvocellular pathway. Thus, these findings were not consistent with the earlier hypothesis that the magnocellular pathway is selectively damaged in glaucoma (Ansari et al., 2002). The variable observations seen between these studies may be due to the complexity and heterogeneity of glaucoma pathogenesis in humans, which would render identification of RGC type susceptibility much more challenging than in animal models. Nonetheless, studies in animal models highlight that the same RGC subtype may respond to injury differently depending on the type of insult, and that ipRGCs and alpha RGCs seem to be more resilient to various types of insult.

It is also worth mentioning that several growth factors and cytokines when delivered exogenously can rescue RGCs to some extent after optic nerve injury. These factors include Bdnf, Cntf, and Igf1 (Kermer et al., 2000; Pernet and Polo, 2006; Yungher et al., 2015; Luo et al., 2016). Along this line, several studies have demonstrated that glial cells including Müller cells release growth factors, which can recue RGCs after axonal injury (Yoo et al., 2021). Thus, a question arises as to, albeit temporarily, why some RGCs are protected while others die when these factors are provided? One possibility is that the receptors for these growth factors are differentially expressed in different RGC types, which would result in preferential survival for those cells that have higher receptor expression. However, RNAseq data (Bray et al., 2019; Tran et al., 2019) show that genes that encode these receptors (e.g., TrkB, Cntf receptor alpha, gp130, IL6 receptor, and Igf1 receptor) are expressed similarly among the different RGC types. Thus, the mechanisms that underlie why some RGC types are more resistant to injury (with or without the supply growth factors) remain elusive.

## 6 Retinal ganglion cells exhibit regional susceptibility to injury

Adding another layer of complexity to RGC death after injury, RGC loss does not always occur uniformly in the retina. In fact, studies have shown that RGC loss can be localized to specific retinal regions during the early stages of injury, indicating that there are regional differences in the susceptibility of RGCs that have not been associated with specific RGC subtypes. For instance, although axotomy triggered RGC death throughout the whole retina, RGC loss was more prominent in the central region of the retina (i.e., close to the optic disk) (Boia et al., 2020). Why RGCs in the peripheral retina are more resistant to damage is currently unclear. A possible explanation could be that since RGCs located in the central retina are right in the area of gaze fixation where they are exposed to more light, they end up experiencing more frequent metabolic stress, “preparing” them with exceptional resistance to stress resulting from an injury (Osborne, 2008; Osborne et al., 2008, 2014). Additionally, since peripheral RGCs have longer intraretinal axons and are likely surrounded by more glial cells (which provide them with trophic and homeostatic support) it could be that they are better capable of coping with an injury than the central RGCs (Büssow, 1980; Stone and Dreher, 1987; Ramírez et al., 2010; Gallego et al., 2012).

The regional difference in vulnerability is highlighted by RGCs that reside in the peripheral ventrotemporal (VT) crescent region of retina. Peripheral VT RGCs were recently identified as the earliest population of RGCs susceptible to ONC injury. As such, they were found to be more vulnerable to degeneration after ONC compared to RGCs residing in other retinal regions (Kingston et al., 2021). In mice, nearly all RGCs in the VT region project to the ipsilateral side of the brain, while RGCs occupying other regions of the retina project to the contralateral side of the brain. The regulatory mechanisms governing ipsilateral RGC specificity, axonal projection, and targeting of retinorecipient areas in the brain are distinguishable from those of contralateral RGC development (Mason and Slavi, 2020). Additionally, some of the molecular mechanisms underlying the selective vulnerability of VT RGCs were recently identified. For example, serotonin transporter (SERT), which regulates refinement of ipsilateral projections and eye-specific segregation in brain targets during postnatal stages, was shown to contribute to higher VT RGC death after ONC (Kingston et al., 2021): SERT expression was significantly upregulated in injured VT axons and loss of SERT induced neuroprotection and axon regeneration of VT RGCs. This SERT-mediated death of injured VT RGCs occurs through activation of integrin β3, which binds to SERT and regulates many of its functions in the nervous system. Additionally, loss of SERT in VT RGCs alters molecular signatures after injury, including transmembrane glycoprotein Nmb (GPNMB).

## 7 Conclusion and future consideration

Injury to the optic nerve results in irreparable death and degeneration of RGCs, disrupting the information highway from the eye to the brain. Much progress has been made in defining RGC types that are resistant (with some also exhibiting a regenerative capacity) and those that are susceptible to injury. However, there are many remaining questions to address. One, what molecular and cellular mechanisms underlie the differential responses to injury? Future studies that are aimed at defining epigenomic, transcriptional as well as protein level differences among the different RGC types using comparative single cell Assay for Transposase-Accessible Chromatin with high-throughput sequencing (ATACseq), scRNAseq and single cell proteomics should reveal more insights into the mechanisms involved. Two, for those RGC types that do survive and regenerate, to what extent can they reconnect with their target cells in the brain? The use of pre-target lesion models (i.e., pre-chiasmic lesion and pre-SC lesion) (Li et al., 2015; Bei et al., 2016) in which the regenerating axons need to travel only short distances, together with transgenic mice in which specific RGC types are labelled should tell us more about whether and how these RGCs reconnect the brain. Additionally, a recent study demonstrated the feasibility of using AAV expressing modified wheat germ agglutinin (WGA) to anterogradely label neurons that are postsynaptic to RGCs. This technique was subsequently combined with scRNASeq, leading to the transcriptomic profiling of RGC-connected SC neurons (i.e., Trans-seq) (Tsai et al., 2022). It remains to be seen if such tools will help us determine whether surviving and regenerating RGCs of specific types reconnect with appropriate SC neurons after injury. Three, it remains a challenge to translate findings in the small animal models to human conditions. As evident above, most animal studies aimed at defining RGC susceptibility were carried out in mice. Since the mouse retina lacks a fovea and comprises significant differences of RGC types compared to the human retina, it remains questionable whether and to what extent the mechanisms that govern survival (or death) of distinct RGC types in mice operate in human RGCs. Incorporating our knowledge of the gene expression, retinorecipient targets, morphological and physiological characteristics exhibited by mouse RGCs (Table 1) may help us identify general mechanisms that either contribute to or inhibit RGC survival and regeneration that can be examined in larger animal models, non-human primates, and ultimately in humans. Importantly, since a given RGC type can respond differently to distinct regenerative strategies, and to distinct types of insults, better understanding of the mechanistic underpinnings of the injury response is critical to identifying an optimal treatment to promote meaningful recovery of vision after injury.

**TABLE 1:** Summary of characteristic features found in resilient and susceptible RGC subtypes.

	Subtype	Somata size	Stratification type	IPL stratification location (S1-S5)	Brain target	Subtype specific markers	RGC type markers
**Resilient**	M1	>M6	monostratified	outer limit of OFF sublamina (S1)	SCN, OPN	Adcyap1+, Opn4+, Nmb-	Opn4, Eomes (Tbr2)
	M2	>M5	monostratified	innermost ON sublamina (S5)	SCN, OPN, SC, dLGN	Spp1+, Tbx20+, Nmb+/-	Opn4, Eomes (Tbr2)
	M3	=M2	bistratified	both ON (S5) and OFF (S1) sublaminas	SC	Cdhr1+, Serpine2+	Opn4, Eomes (Tbr2)
	M4/alpha ON-S	largest of all ipRGCs	monostratified	ON sublamina (S5)	SC, dLGN	Spp1+, ll1rapl2+	Opn4, Spp1, Eomes (Tbr2)
	M5	>M1	monostratified	ON sublamina (S5)	SC, dLGN	Cdhr1+, Serpine2+	Opn4, Eomes (Tbr2)
	M6	smallest of all ipRGCs	bistratified	both ON (S5) and OFF (S1) sublaminas	SC, dLGN	Cdhr1+, Serpine2+	Opn4, Eomes (Tbr2)
	alpha OFF-S	large (20 μm)	monostratified	outer OFF sublamina (S1)	SC, dLGN	Spp1+, Fes+	Spp1, Tbrl
**Susceptible**	W3D2	among smallest of all RGCs	monostratified	center of IPL (S3)	SC, dLGN	Prokr1+	Tusc5
	F-midi-ON	> W3-RGCs	monostratified	center of IPL (S3)	SC, dLGN	Foxp2+, Anza3+	Foxp2
	F-midi-OFF	> W3-RGCs	monostratified	OFF sublamina (S1)	SC, dLGN	Foxp2+, Cdk15+	Foxp2
	F-mini-ON	> W3-RGCs	monostratified	center of IPL (S3)	SC, dLGN	Foxp2+, lrx4+	Foxp2, Tusc5
	F-mini-OFF	> W3-RGCs	monostratified	OFF sublamina (S1)	SC, dLGN	Pde1a+	Foxp2, Tusc5
	F-RGC (Novel)					Rhox5+	Foxp2
	alpha OFF-T	large	monostratified	OFF sublamina (S2)	SC, dLGN	Spp1+, Tpbg+	Spp1
	N-ooDSGCs	smaller than alpha RGCs	bistratified	both ON (S4) and OFF (S2) sublaminas	SC, dLGN, vLGN	Mmp17+	Cartpt, Neurod2
	V/D-ooDSGCs	smaller than alpha RGCs	bistratified	both ON (S4) and OFF (S2) sublaminas	SC, dLGN, vLGN, NOT, MNT	Cartpt+, Col25a1+	Cartpt
	N-RGCs (provisionally)						Neurod2

SC, superior colliculus; dLGN, dorsal lateral geniculate nucleus; vLGN, ventral LGN; SCN, suprachiasmatic nucleus; OPN, olivary pretectal nucleus; MTN, medial terminal nucleus; NOT, nucleus of the optic tract.
